# Drug-coated balloon versus conventional balloon for stent angioplasty in symptomatic intracranial atherosclerotic stenosis

**DOI:** 10.3389/fneur.2025.1686858

**Published:** 2026-01-07

**Authors:** Lili Sun, Chao Chen, Wei Zhao, Yun Song, Meimei Zheng, Hao Yin, Jun Zhang, Yao Meng, Wei Wang, Weili Li, Chenlu Zhu, Ju Han

**Affiliations:** 1 Department of Neurology, The First Affiliated Hospital of Shandong First Medical University, Shandong Provincial Qianfoshan Hospital, Jinan, China; 2 Department of Gerontology, The First Affiliated Hospital of Shandong First Medical University, Shandong Provincial Qianfoshan Hospital, Jinan, China

**Keywords:** drug-coated balloon, intracranial atherosclerotic stenosis, in-stent restenosis, stent angioplasty, stroke

## Abstract

**Background:**

Due to limited evidence on the optimal strategy for intracranial atherosclerotic stenosis (ICAS), further evidence on the safety and efficacy of drug-coated balloon (DCB) predilation stent angioplasty was aimed to be provided.

**Methods:**

Consecutive patients with symptomatic medically refractory ICAS from January 2016 to November 2022 who underwent balloon predilation stent angioplasty were retrospectively analyzed and dichotomized by whether DCB or conventional balloon (CB) was used. The efficacy and safety endpoints were compared by propensity score matching.

**Results:**

A total of 95 patients in the DCB group and 100 patients in the CB group were selected. Of these, 134 patients were matched. The in-stent restenosis (ISR) incidence (1.5% vs. 16.4%, *p* = 0.006) and the median (IQR) stenosis degree at follow-up in the DCB group were significantly lower than those in the CB group [0(0) vs. 0(0), *p* = 0.031]. The primary safety endpoint within 30 days and secondary safety endpoints from 31 days to 1 year were not statistically different between the two groups.

**Conclusion:**

Compared with CB predilation stent angioplasty, DCB predilation stent angioplasty effectively reduced the degree of restenosis and the risk of ISR, but had no advantage in terms of symptomatic restenosis risk currently. However, these findings should be interpreted cautiously because of the aforementioned limitations, and prospective multicenter randomized studies with larger patient number will be required to establish the efficacy and safety of DCB.

## Introduction

Intracranial atherosclerotic stenosis (ICAS) is highly prevalent and is probably the most common cause of stroke in the Asian population (1), as well as being a significant risk factor for stroke recurrence (2). Best medical therapy is the first-line treatment for symptomatic ICAS following the SAMMPRIS trial (3). However, the SAMMPRIS study design might not have contributed to a representative conclusion, and there is accumulating evidence that not all patients with ICAS benefit from best medical treatment alone. Specifically, patients with unstable plaque and hemodynamic ischemic events are at a higher risk for recurrent events (up to 35%) despite medical treatment (4, 5).

Percutaneous transluminal angioplasty, either alone or in combination with stenting, has been regarded as an important complementary treatment for patients who respond poorly to drug therapy (6, 7). Recent randomized controlled trials have shown that balloon angioplasty plus aggressive medical management may be an effective treatment for symptomatic ICAS (8). However, the safety and long-term efficacy of conventionally angioplasty have been challenged by the high incidence of periprocedural complications and follow-up in-stent restenosis (ISR), which accounted for most subsequent recurrence of ischemic events (9). Randomized controlled trials comparing Drug-Eluting Stent(DES) With Bare-Metal Stent (BMS) in Patients With Symptomatic High-grade ICAS found that, compared with BMSs, DESs reduced the risks of ISR and ischemic stroke recurrence in patients with symptomatic high-grade ICAS (10). However, some tortuous vascular anatomies are not suitable for DES.

Drug-coated balloon (DCB) angioplasty is a promising alternative interventional technique (11). Antiproliferative drugs are delivered to the lesion through a catheter and penetrated from balloon surface into the vessel wall to inhibit intimal hyperplasia and prevent restenosis (12). Recent encouraging results from two retrospective cohort studies demonstrated the superiority of DCB compared to conventional balloon (CB) angioplasty and conventional stenting angioplasty in reduce the incidence of restenosis (13, 14). Feared adverse events of the DCB and CB percutaneous transluminal angioplasty technique are early recoil of the stenosis, as well as dissection of the vessel, which would require additional CB percutaneous transluminal angioplasty runs or bailout stenting (13, 15).

There are no publications comparing both the safety and efficacy of DCB and CB for stent angioplasty in ICAS. In this study, both the safety and efficacy of DCB versus CB for stent angioplasty in symptomatic ICAS were assessed.

## Patients and methods

### Study patients

**Figure 1:**
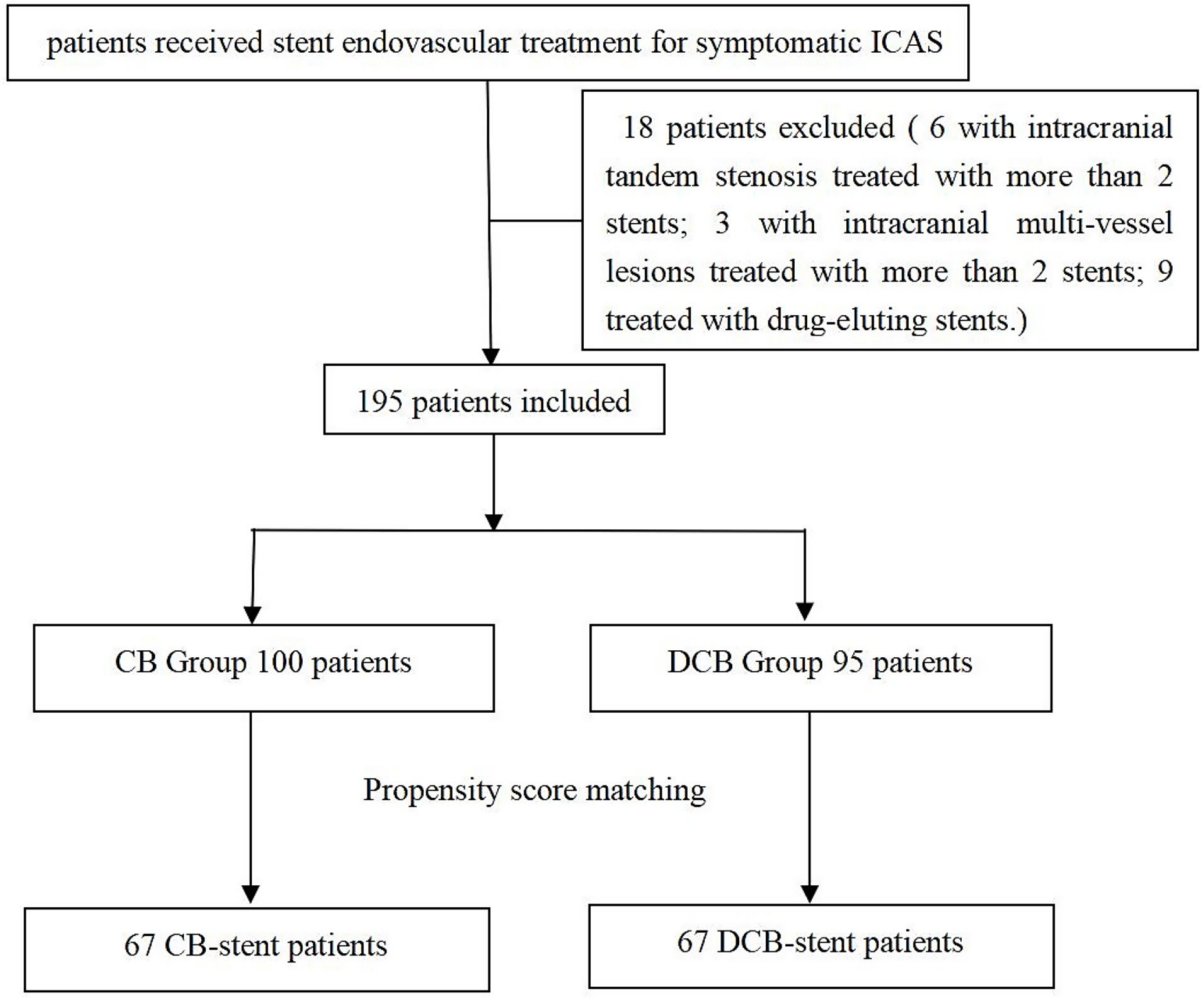
Patient flowchart. ICAS, intracranial atherosclerotic stenosis; CB, conventional balloon; DCB, drug-coated balloon.

The prospective stroke intervention database was retrospectively reviewed to identify patients who had been treated with balloon predilation stent angioplasty for ICAS between January 2016 to November 2022. All the patients had recurrent strokes after aggressive medical management, so stent angioplasty was advised. Only patients with digital subtraction angiography (DSA) or computed tomography angiography (CTA) follow-up outcomes were included in this study. Patients with intracranial tandem stenosis were treated with more than 2 stents; patients receiving treatment for intracranial multi-vessel lesions and patients treated with drug-eluting stents were excluded. The included patients were further divided into DCB group and CB group. All patients were notified of the off-label use of DCB in ICAS. This study was approved by the ethics committee at our hospital. The patient flowchart is illustrated in Figure 1. All research was performed under the relevant regulations and guidelines. All participants gave informed consent.

### Perioperative management and intervention procedure

The details of the interventional treatment have been described previously (13). Prior to the procedure, dual-antiplatelet treatment (DAPT) was combined with 100 mg of aspirin and 75 mg of clopidogrel per day for at least 5 days. The degree of stenosis was determined according to the Warfarin-Aspirin Symptomatic Intracranial Disease (WASID) study (16).

In our center, if the residual stenosis was >50% or there was a vessel dissection after DCB (SeQuent Please; B. Braun, Berlin, Germany) dilation, remedial stenting implantation was performed. For the CB (Gateway; Boston Scientific, Maple Grove, Minnesota) predilation stent angioplasty, the stent was directly implanted after adequate predilation with conventional balloons. The choice of stent in the 2 groups was left to the operator’s discretion, including the balloon-mounted stent (Apollo, MicroPort, Shanghai, China), Wingspan stent system (Boston Scientific, Massachusetts, USA) or Neuroform EZ stent (Boston Scientific, Massachusetts, USA). DAPT was maintained for 6 mo. Device selection depended on lesion morphology, arterial access and vascular characteristics according to the interventionist’s experience.

### Data collection and follow-up outcomes

DSA was recommended for all patients at intervals of 6 months (±1 month) after the index procedure. CTA was conducted instead, if patients refused the DSA. Demographic data, clinical outcomes, periprocedural data, and radiological records as well as other follow-up data were collected and analyzed.

The primary safety endpoint was any causes of stroke include ischemic stroke and intracranial hemorrhage (ICH), acute intraprocedural stent thrombosis (AIST) and transient ischemic attack (TIA) within 30 days. ICH was classified according to the Heidelberg hemorrhage scale (HBC) (17).

The primary efficacy endpoint was ISR after the procedure. ISR was defined as angiographic evidence of >50% stenosis within or immediately adjacent (within 5 mm) to the treated segment. Secondary safety endpoints included any stroke, ischemic stroke, TIA, ICH and all-cause death from 31 days to 1 year. Secondary efficacy endpoints included stenosis degree at follow-up, symptomatic restenosis, and modified Rankin Scale (mRS) score at 1 year. Symptomatic restenosis was defined as previously treated arterial stenosis that recurs over time with ischemic symptoms. The imaging outcomes were reviewed by two investigators.

### Statistical analysis

Statistical analysis was performed using SPSS version 24.0 (IBM, Armonk, New York). Categorical variables were compared using the Fisher’s exact tests or chi-square; continuous variables were analyzed by Mann–Whitney U tests for skewed data or student t-tests for normally distributed data.

Propensity score matching (PSM) was used to match patients in the two groups to eliminate differences in baseline data with demographic data and pre-intervention stenosis as covariates, and nearest-neighbor matching with a caliper value of 0.02 for 1: 1 match. The primary efficacy endpoint was assessed by the Kaplan–Meier method, and the log-rank test was used to test for differences between the groups.

## Results

### General subject characteristics

From January 2016 to November 2022, a total of 195 patients with symptomatic ICAS underwent balloon predilation stent angioplasty, including 95 patients in the DCB group and 100 patients in the CB group. After 1:1 PSM, 134 patients were included in the analysis, with 67 receiving DCB predilation stent angioplasty and 67 receiving CB predilation stent angioplasty. The patient flowchart is illustrated in Figure 1.

### Baseline, periprocedural, and outcome characteristics of the patients

The baseline, periprocedural, and outcome characteristics of the patients are detailed in Table 1. Of the 195 patients, 93.3% completed DSA follow-up. Before PSM, significant differences were observed between the DCB and CB groups regarding stenosis location (anterior circulation: 70.5% vs. 53.0%, *p* = 0.012). A non-significant trend was noted between the DCB and CB groups for pre-treatment stenosis degree (90% vs. 85%, *p* = 0.05) and post-treatment stenosis degree (0 vs. 0, *p* = 0.051). There were no difference in gender, proportion of risk factors, post-treatment mRS score, stent length, stent diameter, or mRS score at one year.

**Table 1:** Baseline characteristics of the patients before PSM.

	DCB group (*n* = 95)	CB group (*n* = 100)	*p* value
Demographics			
Age, mean±SD, years Male, *n* (%)	58.0 ± 9.9 64 (67.4)	58.9 ± 8.9 66 (66.0)	0.516 0.839
Medical history, *n* (%)			
Hypertension Diabetes mellitus Coronary artery disease Hyperlipidemia Atrial fibrillation	61 (64.2) 32 (33.7) 9 (9.5) 3 (3.2) 1 (1.1)	74 (74.0) 36 (36.0) 12 (12.0) 5 (5.00) 1 (1.00)	0.139 0.734 0.569 0.774 1.000
Smoking, *n* (%)	40 (42.1)	47 (47.0)	0.492
Drinking, *n* (%)	44 (46.3)	45 (45.0)	0.854
Stenosis location, *n* (%)			
Anterior circulation Posterior circulation	67 (70.5) 28 (29.5)	53 (53.0) 47 (47.0)	0.012
Admission NIHSS score, median (IQR)	1 (3)	1 (3)	0.773
Stenosis, median (IQR)			
Pre-treatment Post-treatment	90 (15) 0 (0)	85 (10) 0 (0)	0.050 0.051
Stent length, mm	15 (0)	15 (0)	0.086
Stent diameter, mm	3 (1)	3 (0.9)	0.390
Post-treatment mRS score, median (IQR)	1 (1)	1 (1)	0.632
mRS score at 1 year, median (IQR)	1 (1)	1 (1)	0.837

DCB, drug-coated balloon; CB, conventional balloon; NIHSS, National Institutes of Health Stroke Scale; mRS, modified Rankin Scale.

### Key results before PSM

For the primary safety endpoint within 30 days, no significant difference was observed between the DCB and CB groups in terms of any stroke (8.4% vs. 3.0%, *p* = 0.101), ICH (1.1% vs. 1.0%, *p* = 1.000), and AIST (6.3% vs. 7.0%, *p* = 0.848). There were no difference in secondary safety endpoints from 31 days to one year, including any stroke (6.3% vs. 7.0%, *p* = 0.848), ischemic stroke (6.3% vs. 6.0%, *p* = 0.927), TIA (1.1% vs. 2.0%, *p* = 1.000), and ICH (0.0% vs. 1.0%, *p* = 1.000). There were no deaths in either group.

For the primary efficacy endpoint, the DCB group had significantly lower ISR incidence (8.4% vs. 23.0%, *p* = 0.005) compared to the CB group. The indexes of stenosis degree at follow-up was much lower in the DCB group than that in the CB group (0(0) vs. 0(0), *p* = 0.010). There was no significant differences in symptomatic restenosis between the two groups (3.2% vs. 7.0%, *p* = 0.373) (Table 2).

**Table 2:** Safety and efficacy endpoints before propensity score matching.

	DCB group (*n* = 95)	CB group (*n* = 100)	*p* value
Primary safety endpoint within 30 days (%)			
Any stroke Ischemic stroke ICH AIST TIA	8 (8.4) 7 (7.4) 1 (1.1) 6 (6.3) 0 (0)	3 (3.0) 2 (2.0) 1 (1.0) 7 (7.0) 0 (0)	0.101 0.149 1.000 0.848 1.000
Secondary safety endpoints from 31 days to 1 year			
Any stroke Ischemic stroke ICH TIA All-cause death	6 (6.3) 6 (6.3) 0 (0) 1 (1.1) 0 (0)	7 (7.0) 6 (6.0) 1 (1.0) 2 (2.0) 0 (0)	0.848 0.927 1.000 1.000 1.000
Primary efficacy endpoint			
ISR	8 (8.4)	23 (23.0)	0.005
Secondary efficacy endpoints			
Symptomatic restenosis Stenosis degree at follow-up, % mRS score at 1 year, median (IQR)	3 (3.2) 0 (0) 1 (1)	7 (7.0) 0 (0) 1 (1)	0.373 0.010 0.837

DCB, drug-coated balloon; CB, conventional balloon; ICH, intracerebral hemorrhage; AIST, acute intraprocedural stent thrombosis; TIA, transient ischemic attack; ISR, in-stent restenosis; mRS, modified Rankin Scale.

### Key results after PSM

After PSM, each group consisted of 67 patients. The baseline and periprocedural characteristics were well balanced between the two groups.

For the primary safety endpoint within 30 days, no significant difference was observed between the DCB and CB groups in terms of any stroke (10.5% vs. 3.0%, *p* = 0.070), ischemic stroke (9.0% vs. 1.5%, *p* = 0.125), TIA (0% vs. 0%, *p* = 1.000), ICH (1.5% vs. 0%, *p* = 1.000) and AIST (4.5% vs. 7.5%, *p* = 0.727). There were no difference in secondary safety endpoints from 31 days to one year, including any stroke (1.5% vs. 10.5%, *p* = 0.070), ischemic stroke (1.5% vs. 9.0%, *p* = 0.125), ICH (0% vs. 1.5%, *p* = 1.000), and TIA (0% vs. 1.5%, *p* = 1.000). There were no deaths in either group.

**Figure 2:**
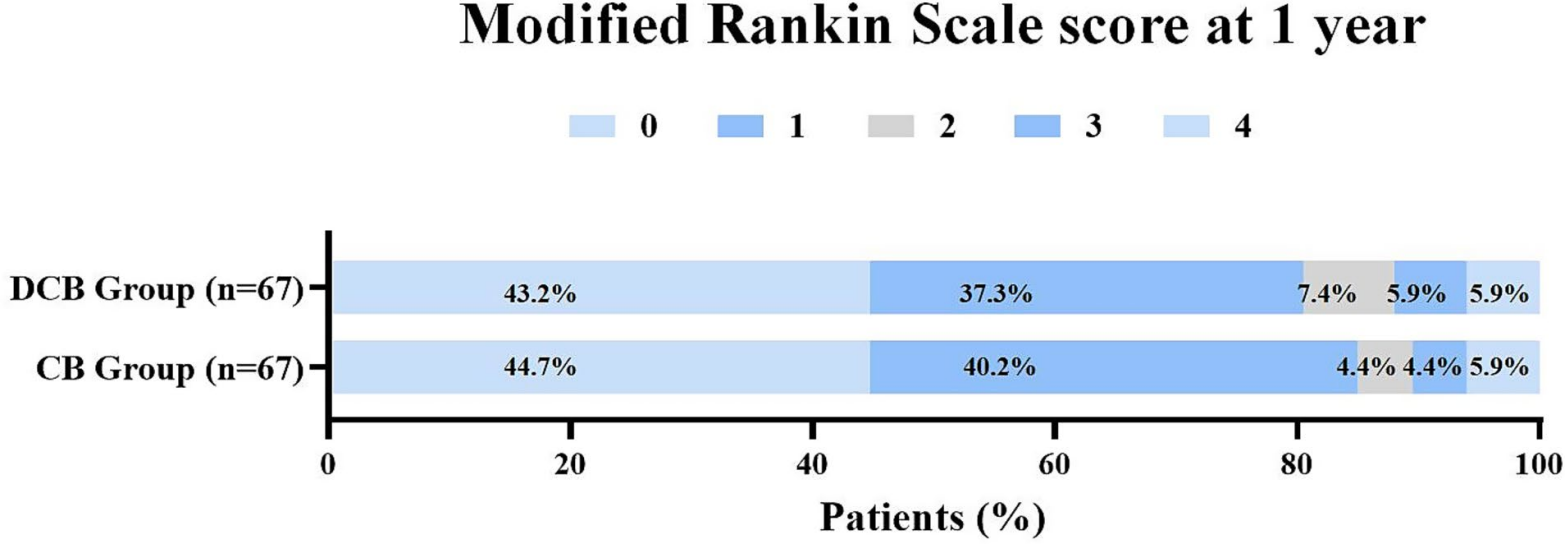
Distribution of global disability at 1 year based on the modifed Rankin scale score.

**Figure 3:**
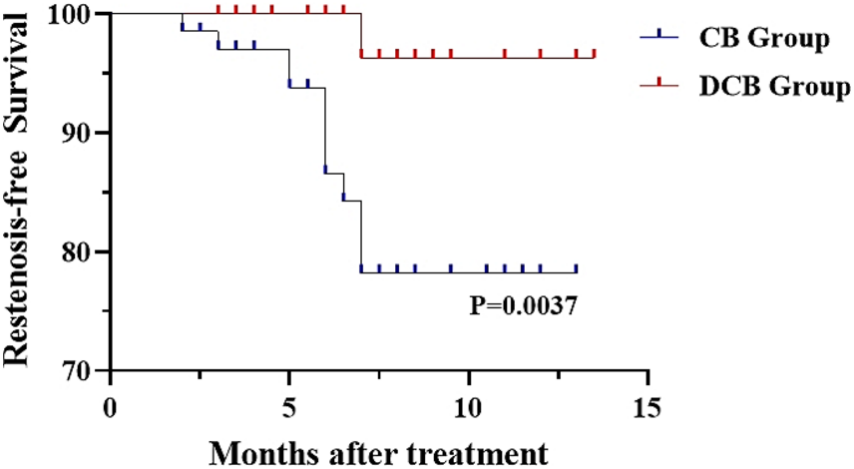
Kaplan–Meier curves of restenosis-free survival probability for DCB and CB patients.

For the primary efficacy endpoint, the DCB group had significantly lower ISR incidence (1.5% vs. 16.4%, *p* = 0.006) compared to the CB group. The indexes of stenosis degree at follow-up was much lower in the DCB group than that in the CB group (0(0) vs. 0(0), *p* = 0.031). There were no significant differences in symptomatic restenosis (0 vs. 6.0%, *p* = 0.375) and mRS score at one year (*p* = 0.654) between the two groups (Table 3). The distribution of global disability at one year based on the mRS score is illustrated in Figure 2. Kaplan–Meier survival analysis showed that patients in the DCB group had a higher restenosis-free survival probability than those in the CB group (*p* = 0.0037) (Figure 3).

**Table 3:** Safety and efficacy endpoints after propensity score matching.

	DCB group (*n* = 67)	CB group (*n* = 67)	*p* value
Primary safety endpoints within 30 days (%)			
Any stroke Ischemic stroke ICH AIST TIA	7 (10.5) 6 (9.0) 1 (1.5) 3 (4.5) 0 (0)	2 (3.0) 1 (1.5) 0 (0) 5 (7.5) 0 (0)	0.070 0.125 1.000 0.727 1.000
Secondary safety endpoints from 31 days to 1 year			
Any stroke Ischemic stroke ICH TIA All-cause death	1 (1.5) 1 (1.5) 0 (0) 0 (0) 0 (0)	7 (10.5) 6 (9.0) 1 (1.5) 1 (1.5) 0 (0)	0.070 0.125 1.000 1.000 1.000
Primary efficacy endpoint			
ISR	1 (1.5)	11 (16.4)	0.006
Secondary efficacy endpoints			
Symptomatic restenosis Stenosis degree at follow-up, median (IQR) mRS score at 1 year, median (IQR)	0 (0) 0 (0) 1 (1)	4 (6.0) 0 (0) 1 (1)	0.375 0.031 0.654

DCB, drug-coated balloon; CB, conventional balloon; ICH, intracerebral hemorrhage; AIST, acute intraprocedural stent thrombosis; TIA, transient ischemic attack; ISR, in-stent restenosis; mRS, modified Rankin Scale.

## Discussion

In this study, both the safety and therapeutic efficacy of DCB and CB for stent angioplasty in symptomatic ICAS were compared. The findings suggest that, after DCB dilation, the median stenosis degree and ISR incidence at 6-month follow-up were significantly lower than those observed with CB predilation stent angioplasty. However, there was no difference in symptomatic restenosis between the two groups. The goal of angioplasty and stenting is to restore optimal blood flow in narrowed arteries. ISR has hindered its long-term effects in coronary, peripheral, and intracranial artery interventional treatments. For intracranial vasculature, the ISR rate may be as high as 32.4% for bare metal stents (18).

With the development of interventional devices, DCB has emerged as a potential treatment for endovascular therapy. Coated with antiproliferative drugs, DCB can effectively inhibit intimal hyperplasia. It is increasingly recognized that DCB is superior for some high-risk patients with coronary and peripheral artery disease (19, 20). In tortuous neurovascular anatomy, DCB is more flexible due to the softer distal tip. Additionally, DCB offers a uniform drug coverage of the diseased vessel lumen (11).

For intracranial vasculature, most studies on DCB have been case series focusing on the treatment of ISR (21, 22). Recently, more researches have focused on comparing DCB with conventional stent angioplasty or CB percutaneous transluminal angioplasty. In our previous study, DCB was systematically compared with commonly used clinical stent systems, including balloon-mounted stents and self-expanding stents. The results showed that the median stenosis degree and restenosis rate at follow-up were significantly lower in the DCB group compared to the non-DCB group (13). Meanwhile, Tang et al. compared DCB with CB percutaneous transluminal angioplasty in symptomatic ICAS and found that DCB angioplasty effectively reduced the incidence of restenosis (14). The latest meta-analysis suggests that DCB angioplasty may be safer and more effective than stent angioplasty for Intracranial atherosclerotic disease, with lower rates of restenosis and ischemic complications (23).

However, the mechanical support provided by the balloon is thought to be weaker than that of a stent. If residual stenosis was greater than 50% or if vessel dissection occurred after DCB or CB dilation, remedial stent implantation was necessary. Currently, there are no publications assessing DCB versus CB for stent angioplasty in symptomatic ICAS. Therefore, the rationality behind the series was to combine the procedural safety and technical simplicity of stent angioplasty with the beneficial effects of paclitaxel on ISR rates by predilation using DCB. The results are encouraging. Our results support the findings that the median stenosis degree and ISR rate at follow-up were significantly lower in the DCB group compared to the CB group. The relatively high rate of restenosis (16.42%) in our conventional stent system group aligns with previous reports of 24.5–32.4% (9, 18). The stenosis was very similar in both groups after treatment, although it was lower in the DCB group at 6-month follow-up. This suggests that DCB may have a positive remodeling effect on the vascular lumen, which may be related to improved proliferation and distribution of smooth muscle cells. Although there were no statistical differences, the absolute incidence of symptomatic restenosis and recurrent ischemic stroke was lower in the DCB group compared to the CB group. This may be partly associated with the short follow-up period and small sample size.

DCB may be a promising option for treating patients with ICAS because it has the potential to minimize peri-interventional and long-term complication rates in endovascular treatment (11). In our study, the restenosis rate of DCB predilation stent angioplasty was 1.49% lower than previous reports of DCB predilation stent angioplasty with a self-expanding stent (Enterprise) (24). This may be due to the advantages of DCB and the more flexible choice of interventional materials. The development of these balloons will be very important in the near future. The currently available drug-eluting coronary stents are not suitable for these lesions due to their stiffness, whereas the use of sufficiently flexible self-expanding bare-metal stents results in high ISR rates (25). Combining flexible stents with DCB can be safe and efficient in treating these lesions.

### Limitations

Our study has some limitations. First, the limitations of the data from retrospective clinical series, although propensity score matching analysis was applied to balance potential confounders, were unlikely to match the rigor of randomized controlled trials. Second, treatment choices based on the interveners’ experience and the patients’ preferences might lead to selection bias. Third, the matched sample size was not large enough, the imaging follow-up was scheduled for 6 months, lacking long-term follow-up results. Additionally, these data are from a single experienced high-volume center and may not be generally applicable.

## Conclusion

In this single-center study of propensity-matched patients with symptomatic severe ICAS, DCB predilation stent angioplasty effectively reduced the degree of restenosis and the risk of ISR, but had no advantage in terms of symptomatic restenosis risk. This study adds important insights to the existing clinical evidence for ICAS treatment strategies. However, these findings should be interpreted cautiously because of the aforementioned limitations, and prospective multicenter randomized studies with larger patient number will be required to establish the efficacy and safety of DCB.

## Data Availability

The original contributions presented in the study are included in the article/supplementary material, further inquiries can be directed to the corresponding author.
